# The challenge of the COVID-19 pandemic: what can we learn from history?

**DOI:** 10.1590/1677-5449.200209

**Published:** 2021-03-19

**Authors:** Vanessa Prado dos Santos

**Affiliations:** 1 Universidade Federal da Bahia – UFBA, Instituto de Humanidades Artes e Ciências Professor Milton Santos – IHAC, Salvador, BA, Brasil.

History is an essential element of our professional training. At medical school, we learn to investigate patients’ clinical history, valuing the history of the current complaint, previous pathological history, and family history. We must know the natural history of disease underlying the most varied range of pathologies. In vascular surgery, the history of trauma medicine and organ transplantation are important for us to learn about the operating techniques we are employing. These examples show that when we think about the challenges faced in daily medical practice, history is our ally. At the moment, we are challenged by the Coronavirus Disease 2019 (or COVID-19) pandemic. Up to the end of September 2020, 33,249,563 cases and 1,000,040 deaths had been confirmed worldwide.^1^ In Brazil, 4,732,309 cases and 141,741 deaths had been confirmed.^1^ To better comprehend the dimensions of these numbers, 1,316,179 deaths were recorded in 2018 in Brazil, 357,770 caused by cardiovascular diseases, 155,191 by diseases of the respiratory system, and 54,679 by infectious and parasitic diseases.^2^ These data illustrate the impact of the Covid-19 pandemic in Brazil, since the number of deaths up to September is similar to the total mortality due to all respiratory diseases in 2018.^1^ However, exact and health sciences are not the only disciplines to help us to deal with this challenge. What does history have to say about previous pandemics that afflicted the world?

Infectious diseases have affected humanity for thousands of years. More than a thousand years before Christ (BC), it is probable that Egypt was hit by major epidemics.^3^ The Greek and Roman empires also suffered from contagious diseases.^3^ Hippocrates’ studies of diseases date from more than 300 years BC.^4^ There are reports from 500 BC in India about sickness and death from symptoms compatible with cholera.^5^

In the XIV century, from 1347 to 1352, the black death (or bubonic plague) dominated Europe and it is estimated that it was responsible for killing around one quarter of the population.^5^ At that time, society did not yet understand what caused the disease nor how it was transmitted, which provided fertile ground for violence, persecution, and prejudice.^5^ The situation in Europe in the XIV century was highly favorable to dissemination of the black death, transmitted by rodent fleas.^5^ Overcrowding among the inhabitants of urban centers, without the most basic infrastructure for hygiene, subject to climatic extremes, hunger, and wars provided the ideal environment for propagation of the disease.^5^ The bacteria responsible, *Yersinia Pestis*, was identified in 1894 and was named in homage to the researcher who discovered it, the scientist Alexandre Yersin.^3^^,^ 5 The black death reappeared in many different countries at other times in history. The city of Porto, in Portugal, went through a black death epidemic in 1899, with imposition of restrictive measures such as isolation, quarantine, and obligatory bathing that were very unpopular with residents.^6^

Over the years, society has questioned the cause of diseases, which were commonly associated with improper behavior and/or as a type of punishment. In the nineteenth century, two hypotheses had their supporters/defenders and antagonists.^3^ The miasma or miasmatic theory proposed that emanations of air and the atmosphere contaminated with bad odors were responsible for spreading diseases.^3^^,^ 6 ^,^ 7 Contradicting this theory were the contagionists, who advocated the theory of contagion by germs, claiming that microorganisms invisible to the human eye was transmitted from the sick to the healthy.^3^ The contagion theory was formulated in 1546 by Girolamo Fracastoro but was contested and ignored for many decades.^3^

In the nineteenth century, 1817, Europe went through a cholera pandemic, considered a global disease in 1830, and which reappeared around 40 times from 1831 to 1912.^5^ In the cholera epidemics of 1831 and 1848, more than 70 thousand lives were lost in England and Wales.^8^ In 1854, the city of London had high demographic density, with many people living together in small rooms and had problems with disposal of refuse and sewage, which were collected in pits spread around the city.^8^ Even before *Vibrio cholerae* identification, the doctor John Snow, who is considered the first epidemiologist, used statistical methods to formulate a hypothesis about the way cholera was transmitted, helping to weaken the miasma theory.^9^ John Snow studied and mapped the incidence of cholera in different locations in London, proving that it was transmitted by contaminated water.^3^^,^ 9 Also in London, the hot and dry summer of 1858 triggered an episode known as “the great stink”, during a time in which many people believed in the miasma theory.^10^ The fetid and unpleasant air convinced the English of the need for sanitation works in the city. The English parliament approved construction of a sewage system, from 1859 to 1875, which took the city off the cholera map.^10^ Growing evidence of microorganisms and about contagion of diseases and the discoveries of the French researcher Louis Pasteur culminated in 1857, considered the start of Microbiology.^3^^,^ 11 Pasteur’s studies explained fermentation, challenged the theory of spontaneous generation and, in conjunction with Robert Koch’s identification and isolation of the bacillus responsible for tuberculosis, in 1882, confirmed the role of microorganisms as the etiologic agents of diseases..^3^^,^ 9 ^,^ 10 ^,^ 12

However, the microscopes and new techniques for identification and classification of countless bacterial species did not prevent new pandemics. Viral diseases brought new challenges, with epidemics of smallpox and yellow fever. Viruses were studied by Dimitri Ivanovsky in 1892 and by Martinus Beijerinck in 1898 in the tobacco mosaic disease, who defined them as filterable submicroscopic agents.^3^^,^ 13 In 1918, Spanish flu found a population impacted by the First World War, and became responsible for about the deaths of 50 million people.^6^^,^ 14 ^,^ 15 This flu’s name is a cause of controversy. It is probable that the epidemic began in the United States, where, considering all three waves of the disease, around 675,000 lives were lost.^7^^,^ 14 ^-^ 16 More recently, genetic studies of preserved genetic material identified the *Influenza A* virus, H1N1, as the subtype responsible for the pandemic of 1918.^17^ Subtypes H2N2 and H3N2 caused pandemics in 1957 and 1968, respectively.^16^^,^ 18 In general, the many different epidemics went through periods of ignorance, negation, incomprehension, and negotiation between social groups, arriving at periods of adoption of more or less restrictive preventative measures, and stimulating scientific progress.^6^^-^ 8 ^,^ 14 ^,^ 15 Technological advances contributed to management of epidemics, with the arrival of antibiotics, antivirals, and equipment, in addition to many other new technologies .

Locating Brazil in the panorama of epidemics, it is estimated that cholera caused the deaths of 200,000 people from 1855 to 1856.^5^ In the twentieth century, the country went through its seventh cholera pandemic, from 1991 to 1996, with more than 150 thousand notified cases.^19^ Unhealthy conditions in Brazilian cities favored epidemics of smallpox and yellow fever, causing impacts on maritime trade and on the population’s economic status.^14^^,^ 20 In 1904, adoption of coercive measures including obligatory vaccination against smallpox led to a popular protest known as the Vaccine Revolt.^20^ The Spanish flu arrived in Brazil in September 1918, finding the country in a scenario of financial crisis, political conflict, poverty, and poor sanitation.^21^ The literature describes the impact of the flu on the cities of Rio de Janeiro, Salvador, and Campinas.^7^^,^ 14 ^,^ 21 In Rio de Janeiro, it is estimated that the pandemic caused around 15,000 deaths, with greater morbidity and mortality in areas with deficient sanitary infrastructure.^7^

There is a need to take a broader view on the apparently democratic nature of epidemics.^14^ Over the course of history, poor living conditions, hunger, unhealthy housing, bad refuse management, a lack of sewage, and absence of water treatment have been conditions that favor major epidemics.^3^^,^ 14 ^,^ 21 In Brazil, data published in 2017 show that 61.4% of the population were connected to a sewage system and 42.6% to a system that provided both collection and treatment, while the country’s Southeast region was the only one in which more than half of the population have sewage treatment.^22^

We are part of a constantly evolving world.^13^ It is estimated that there is a larger number of viral particles in the biosphere than the total number of cells.^13^ The brief historical retrospective in this editorial is not intended to present a detailed chronology or exhaust the subject of epidemics that have marked the history of humanity. The text is intended to describe some historical events, contributing to reflections on the different paths that we can find to face the challenge of this pandemic, which consider the dynamic interaction between man and the environment, which reflect on solidarity and life in community, and which build a dialogue on an interdisciplinary proposal, in which the physical sciences, the humanities, and the biological, social, and environmental sciences all cooperate to achieving better quality of life for all of us.

## References

[1] World Health Organization (2020). WHO Coronavirus Disease (COVID-19) Dashboard.

[2] Brasil (2020). Mortalidade - Brasil.

[3] Opal SM (2009). A brief history of microbiology and immunology. Vaccines. Biography.

[4] Berlinger GA (1988). Doença..

[5] Lewisohn R (2003). Três epidemias: lições do passado..

[6] Almeida MAP (2012). O Porto e as epidemias: saúde e higiene na imprensa diária em períodos de crise sanitária, 1854-56, 1899, 1918. Rev Hist Soc Cult..

[7] Goulart AC (2005). Revisitando a espanhola: a gripe pandêmica de 1918 no Rio de Janeiro. Hist Cienc Saude Manguinhos.

[8] Johnson S (2008). O mapa fantasma: como a luta de dois homens contra o cólera mudou o destino de nossas metrópoles..

[9] Cerda LJ, Valdivia CG (2007). John Snow, la epidemia de cólera y el nacimiento de la epidemiología moderna. Rev Chilena Infectol.

[10] Halliday S (2001). Death and miasma in Victorian London: an obstinate belief. BMJ.

[11] Schwartz M (2001). The life and works of Louis Pasteur. J Appl Microbiol.

[12] Akkermans R (2014). Robert Heinrich Herman Koch. Lancet Respir Med.

[13] Domingo E, Domingo E (2020). Introduction to virus origins and their role in biological evolution.. Virus as populations: composition, complexity, quasispecies, dynamics, and biological implications..

[14] Bertucci-Martins LM (2005). Memória que educa: epidemias do final do século XIX e início do XX. Educ Rev.

[15] Jester B, Uyeki T, Jernigan D (2018). Readiness for responding to a severe pandemic 100 years after 1918. Am J Epidemiol.

[16] Glezen WP (1996). Emerging Infections: Pandemic Influenza. Epidemiol Rev.

[17] Reid AH, Fanning TG, Hultin JV, Taubenberger JK (1999). Origin and evolution of the 1918 “Spanish” influenza virus hemagglutinin gene. Proc Natl Acad Sci USA.

[18] Kilbourne ED (2006). Influenza Pandemics of the 20th Century. Emerg Infect Dis.

[19] Gerolomo M, Penna MLF (1999). Os primeiros cinco anos da sétima pandemia de cólera no Brasil. Inf Epidemiol SUS..

[20] Hochman G (2011). Vacinação, varíola e uma cultura da imunização no Brasil. Cien Saude Colet.

[21] Souza CMC (2005). A gripe espanhola em Salvador, 1918: cidade de becos e cortiços. Hist Cienc Saude Manguinhos.

[22] Brasil (2017). Atlas esgotos: despoluição de bacias hidrográficas..

