# Supplementary material for: The challenge of the COVID-19 pandemic: what can we learn from history?
Source: J Vasc Bras. 2021 Mar 19;20:e20200209. doi: 10.1590/1677-5449.200209 (PMC8147886; doi:10.1590/1677-5449.200209)
Supplement: Translation

# Translation: O desafio da pandemia da COVID-19: o que podemos aprender com a história?

**Authors:** Vanessa Prado dos Santos

**Affiliations:** 1 Universidade Federal da Bahia – UFBA, Instituto de Humanidades Artes e Ciências Professor Milton Santos – IHAC, Salvador, BA, Brasil.

A história é um elemento essencial na nossa formação profissional. Na graduação, aprendemos a investigar a história clínica, na anamnese, valorizando a história da moléstia atual, a história patológica pregressa e a história familiar. Sobre as diversas patologias, precisamos conhecer a história natural da doença. Na cirurgia vascular, as histórias do trauma e do transplante de órgãos são importantes para aprendermos sobre as técnicas operatórias que empregamos. Os exemplos mostram que, para atuar nos desafios que se impõem ao cotidiano da Medicina, temos a história como aliada. Neste momento, nos vemos desafiados pela pandemia da *Coronavirus Disease* 2019, ou COVID-19. No mundo todo, até o final de setembro de 2020, foram confirmados 33.249.563 casos e 1.000.040 óbitos^1^. No Brasil, foram 4.732.309 casos confirmados e 141.741 mortes^1^. Buscando compreender melhor a dimensão desses números, foram registrados no Brasil, em 2018, 1.316.179 óbitos, sendo 357.770 por doenças do aparelho circulatório, 155.191 por doenças do aparelho respiratório e 54.679 por doenças infecciosas e parasitárias^2^. Os dados mostram o impacto da pandemia da COVID-19 no Brasil, sendo o número de mortes, até setembro, semelhante ao total de mortes por todas as doenças respiratórias em 2018^1^. No entanto, as ciências exatas e as da saúde não são as únicas a nos ajudar nesse momento desafiador. O que será que a história nos conta sobre as pandemias que atingiram o mundo?

As doenças infecciosas afetam a humanidade há milhares de anos. Mais de mil anos Antes de Cristo (AC), é provável que grandes epidemias tenham atingido o Egito^3^. Os impérios Grego e Romano também sofreram com as doenças contagiosas^3^. Datam de mais de 300 anos AC os estudos de Hipócrates sobre as doenças^4^. Na Índia, 500 anos AC, encontram-se relatos sobre o adoecimento e morte por sintomas compatíveis com a cólera^5^.

No século XIV, entre 1347 e 1352, a peste negra, ou bubônica, dominou o continente europeu, estimando-se que tenha causado a morte de cerca de um quarto da população^5^. A sociedade da época ainda não compreendia qual a causa, nem como se dava a transmissão da doença, o que configurava um terreno fértil para a violência, a perseguição e o preconceito^5^. O cenário europeu do século XIV era amplamente favorável à disseminação da peste negra, transmitida através das pulgas dos roedores^5^. A aglomeração dos cidadãos nos centros urbanos, sem estrutura mínima de higiene, sujeitos às intempéries climáticas, à fome e às guerras, forneceram o ambiente ideal para a propagação daquela doença^5^. A bactéria *Yersinia Pestis* foi identificada em 1894, recebendo esse nome em homenagem ao seu pesquisador, o cientista Alexandre Yersin^3^^,^ 5. A peste negra reapareceu em diversos países e em outros momentos da história. A cidade do Porto, em Portugal, passou por uma epidemia de peste negra em 1899, com o estabelecimento de medidas restritivas, como isolamento, quarentena e banhos obrigatórios, muito impopulares entre os moradores^6^.

Durante os anos, a sociedade se interrogou a respeito da causa das doenças, sendo comumente associadas a comportamentos impróprios e/ou a uma forma de punição. No século XIX, duas hipóteses contaram com defensores e antagonistas^3^. A teoria miasmática, ou dos miasmas, propunha que emanações do ar, da atmosfera contaminada com maus odores, eram responsáveis pela disseminação das doenças^3^^,^ 6 ^,^ 7. Contrariando essa teoria estavam os contagionistas, defensores da teoria do contágio dos germes, afirmando que um microrganismo, invisível aos olhos humanos, era transmitido dos doentes aos não doentes^3^. Girolamo Fracastoro, em 1546, formulou a teoria do contágio, que foi combatida e ignorada durante várias décadas^3^.

Ainda no século XIX, em 1817, a Europa passou por uma pandemia de cólera, doença considerada global em 1830, e que reapareceu cerca de 40 vezes entre 1831 e 1912^5^. A Inglaterra e o País de Gales perderam, nas epidemias de cólera de 1831 e 1848, mais de 70 mil vidas^8^. Em 1854, a cidade de Londres tinha uma elevada densidade demográfica, com dezenas de pessoas vivendo aglomeradas em pequenos cômodos, e dificuldade na remoção do lixo e dos dejetos, que eram recolhidos nas fossas espalhadas pela cidade^8^. Mesmo antes da identificação do *Vibrio cholerae*, o médico John Snow, considerado o primeiro epidemiologista, utilizou métodos estatísticos para formular a hipótese sobre a forma de transmissão da doença, ajudando a enfraquecer a teoria miasmática^9^. John Snow estudou e mapeou a incidência de cólera em diferentes localidades de Londres, demonstrando sua transmissão através da água contaminada^3^^,^ 9. Ainda em Londres, o verão quente e seco de 1858 deflagrou o episódio conhecido como o grande fedor (*the great stink*), em uma época em que a teoria miasmática contava com muitos adeptos^10^. O ar fétido e desagradável convenceu os ingleses da necessidade de ações de saneamento na cidade. O parlamento inglês aprovou a construção de um sistema de esgotos, entre 1859 e 1875, que tirou a metrópole do mapa da cólera^10^. Evidências crescentes a respeito dos microrganismos, do contágio das doenças e as descobertas do pesquisador francês Louis Pasteur conduziram ao ano de 1857, considerado o início da Microbiologia^3^^,^ 11. As pesquisas de Pasteur explicaram a fermentação, combateram a teoria da geração espontânea e, somadas à identificação e ao isolamento, por Robert Koch, do bacilo causador da tuberculose, em 1882, confirmaram o papel dos microrganismos como agentes etiológicos de diversas doenças. 3 ^,^ 9 ^,^ 10 ^,^ 12.

Os microscópios, as técnicas de identificação e a classificação de inúmeras espécies de bactérias não impediram que outras pandemias chegassem às comunidades. As doenças virais trouxeram novos desafios, com epidemias de varíola e febre amarela. Os vírus foram estudados por Dimitri Ivanovsky em 1892, e por Martinus Beijerinck em 1898 na doença do mosaico do tabaco, tendo sido definidos como agentes filtráveis submicroscópicos^3^^,^ 13. No ano de 1918, a gripe espanhola encontrou a população sob o impacto da Primeira Guerra Mundial, sendo responsável pela morte de cerca de 50 milhões de pessoas^6^^,^ 14 ^,^ 15. O nome da gripe é motivo de controvérsia. É provável que a epidemia tenha se iniciado nos Estados Unidos, onde, considerando-se as três ondas da doença, ceifou cerca de 675.000 vidas^7^^,^ 14 ^-^ 16. Mais recentemente, através do estudo genético de tecido preservado, o vírus *Influenza A*, H1N1, foi identificado e considerado o subtipo responsável pela pandemia de 1918^17^. Os subtipos H2N2 e H3N2 causaram pandemias em 1957 e 1968, respectivamente^16^^,^ 18. De maneira geral, as diversas epidemias passaram por períodos de desconhecimento, negação, incompreensão e negociação entre os grupos sociais, chegando a momentos de adoção de medidas preventivas, mais ou menos restritivas, estimulando o progresso da ciência^6^^-^ 8 ^,^ 14 ^,^ 15. Os avanços tecnológicos trouxeram contribuições ao enfrentamento das epidemias, com a chegada dos antibióticos, antivirais e equipamentos, entre muitos outros progressos.

Localizando o Brasil no panorama das epidemias, estima-se que a cólera tenha causado o óbito de 200.000 pessoas entre 1855 e 1856^5^. No século XX, o país passou pela sétima pandemia de cólera, entre os anos de 1991 e 1996, com mais de 150 mil casos notificados^19^. Epidemias de varíola e febre amarela foram favorecidas pelas condições insalubres das cidades brasileiras, tendo impacto no comércio marítimo e na vida econômica da população^14^^,^ 20. Em 1904, a adoção de medidas coercitivas, que incluíam a obrigatoriedade da vacinação contra varíola, levou ao protesto popular conhecido como Revolta da Vacina^20^. A gripe espanhola chegou ao Brasil em setembro de 1918, encontrando um cenário de crise financeira, conflitos políticos, pobreza e estrutura sanitária precária^21^. A literatura relata o impacto da gripe nas cidades do Rio de Janeiro, Salvador e Campinas^7^^,^ 14 ^,^ 21. No Rio de Janeiro, estima-se que a pandemia tenha causado em torno de 15.000 óbitos, com maior morbimortalidade nas áreas com estrutura sanitária deficiente^7^.

A aparente democracia das epidemias carece de um olhar mais atento^14^. Ao longo da história, más condições de vida, fome, moradias insalubres, acúmulo de lixo, ausência de esgotamento sanitário e de tratamento da água foram condições favoráveis às grandes epidemias^3^^,^ 14 ^,^ 21. No Brasil, dados publicados em 2017 mostraram que 61,4% da população contavam com rede coletora de esgotos e 42,6% com coleta e tratamento, sendo a região Sudeste a única em que o tratamento dos esgotos chegava a mais da metade da população^22^.

Fazemos parte de um mundo em constante evolução^13^. Estima-se que, na biosfera, haja um número maior de partículas virais do que o total de células^13^. A breve retrospectiva histórica deste editorial não tem a intenção de apresentar uma cronologia detalhada ou de esgotar o tema a respeito das epidemias que marcaram a história da humanidade. O texto pretende trazer alguns acontecimentos históricos, contribuindo para a reflexão sobre os diferentes caminhos que podemos encontrar para enfrentar os desafios dessa pandemia, que considerem a interação dinâmica entre o homem e o meio ambiente, que pensem a solidariedade e a vida em comunidade e que dialoguem sobre uma proposta interdisciplinar, na qual as ciências exatas, humanas, biológicas, sociais e ambientais colaborem para uma melhor qualidade de vida para todos nós.
